# Migraine to Mesenteric Mishap: A Case Report of Ischemic Colitis Due to Sumatriptan

**DOI:** 10.7759/cureus.102093

**Published:** 2026-01-22

**Authors:** Michael G Quon, Chanthel Kokoy-Mondragon, Cameron M Quon

**Affiliations:** 1 Department of Medicine, Vatche and Tamar Manoukian Division of Digestive Diseases, David Geffen School of Medicine, University of California, Los Angeles, USA; 2 Department of Internal Medicine, Keck School of Medicine, University of Southern California, Los Angeles, USA

**Keywords:** abdominal pain, colitis, ischemic colitis, medication-induced colonic ischemia, migraine headache, non-steroidal anti-inflammatory drug, subtotal colectomy with ileostomy, sumatriptan, vasoconstriction

## Abstract

Ischemic colitis (IC) results from reduced blood flow to the colon, which can lead to tissue infarction and may be precipitated by medications that induce vasoconstriction. This case involves a 73-year-old female with a history of migraines and chronic back pain who presented with abdominal pain and was diagnosed with severe IC, ultimately requiring subtotal colectomy and ileostomy. The patient had been using nonsteroidal anti-inflammatory drugs for many years, then initiated high doses of the 5-hydroxytryptamine receptor agonist sumatriptan prior to her first episode of IC, suggesting that sumatriptan was the primary etiology. This case represents the patient’s third episode of IC, highlighting the importance of thorough medication review in identifying potential etiologies.

## Introduction

Ischemic colitis (IC) represents the most common form of ischemic injury affecting the gastrointestinal tract [1]. IC results from reduced or interrupted colonic blood flow, with clinical severity ranging from transient, self-limited ischemia confined to the mucosa and submucosa to fulminant transmural infarction [2]. The actual incidence of IC remains underestimated because mild and transient cases are frequently difficult to diagnose, owing to their acute and self-limiting nature [3]. IC development is associated with any condition or exposure that reduces colonic perfusion, including cardiovascular disease, hematological disorders, microvascular disease, and the use of certain medications or drugs [4].

Medications associated with IC include oral contraceptives, vasoconstrictors, psychotropics, interferon-alpha, cocaine, nonsteroidal anti-inflammatory drugs (NSAIDs), 5-hydroxytryptamine-3 (5-HT_3_) receptor antagonists, and 5-hydroxytryptamine-1 (5-HT_1_) receptor agonists [4,5]. Sumatriptan is a 5-HT_1_ receptor agonist used for treating migraine headaches; however, while the vasoconstrictive properties of the drug are intended for cranial vessels, they may have systemic effects on mesenteric circulation and are thus a rare cause of IC, with only a limited number of published case reports [5-12].

## Case presentation

A 73-year-old woman with a history of migraine headaches presented to the emergency room with an acute onset of severe abdominal pain. She had been in her usual state of health when the pain abruptly caused her to wake from sleep at midnight. The pain was sharp, localized to the lower abdomen, persistent, and unrelieved by a normal soft bowel movement. Associated symptoms included nausea and one episode of vomiting, with no fever, chest pain, shortness of breath, or urinary symptoms.

The patient presented in decompensated shock with hypotension (blood pressure: 58/33 mmHg), tachycardia (heart rate: 100 beats/min), and tachypnea (respiratory rate: 24 breaths/min). While the etiology of shock was initially unclear and global hypoperfusion was considered, there was immediate concern for regional ischemia given her lower abdominal tenderness without rebound, hypoactive bowel sounds, and history of similar symptoms during a prior episode of IC.

Initial laboratory findings were largely unremarkable except for lactic acidosis with a borderline anion gap metabolic acidosis (Table 1). She responded promptly to a bolus of isotonic intravenous (IV) fluids, with subsequent normalization of blood pressure and serum lactate levels. Given the concern for an ischemic process and possible sepsis, broad-spectrum antimicrobial therapy was initiated, including IV cefepime (2 g every 12 h), meropenem (1 g every 8 h), micafungin (100 mg every 24 h), and linezolid (300 mg every 12 h).

**Table 1:** Laboratory findings on presentation. WBC: white blood cell count; BUN: blood urea nitrogen; Na: sodium; K: potassium; Cl: chloride; HCO_3_: bicarbonate; ALP: alkaline phosphatase; AST: aspartate aminotransferase; ALT: alanine aminotransferase

Parameter	Result	Units	Reference range
WBC	8.6	K/uL	4.5-11.0
Hemoglobin	14.1	g/dL	12.0-16.0
Hematocrit	43.6	%	37.0-47.0
Platelet count	337	K/uL	140-400
Na	141	mmol/L	138-146
K	4.4	mmol/L	3.5-4.9
Cl	103	mmol/L	98-109
HCO_3_	21	mmol/L	24-29
Anion gap	17	mmol/L	8-19
Serum creatinine	0.8	mg/dL	0.6-1.2
BUN	30	mg/dL	7-20
ALP	58	U/L	35-105
AST	26	U/L	<33
ALT	20	U/L	<34
Lipase	57	U/L	13-60
Lactate	3.1	mmol/L	0.5-2.0

**Figure 1:**
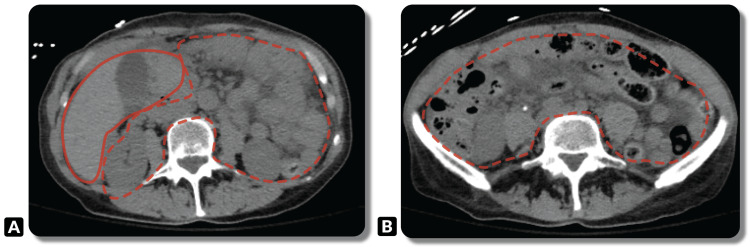
Axial CT angiogram images at two different levels. (A) Portal edema (solid red line) with diffuse mesenteric stranding and edema (dashed red line). (B) Diffuse mesenteric stranding and edema (dashed red line) at a different axial level.

A gastroenterologist was consulted early in the clinical course, and the patient underwent a computed tomography (CT) angiogram, which demonstrated diffuse mesenteric stranding and edema (Figure 1). Angiography was performed to additionally assess vascular patency, which was unremarkable. Colonic diverticulosis was identified without evidence of diverticulitis.

**Figure 2:**
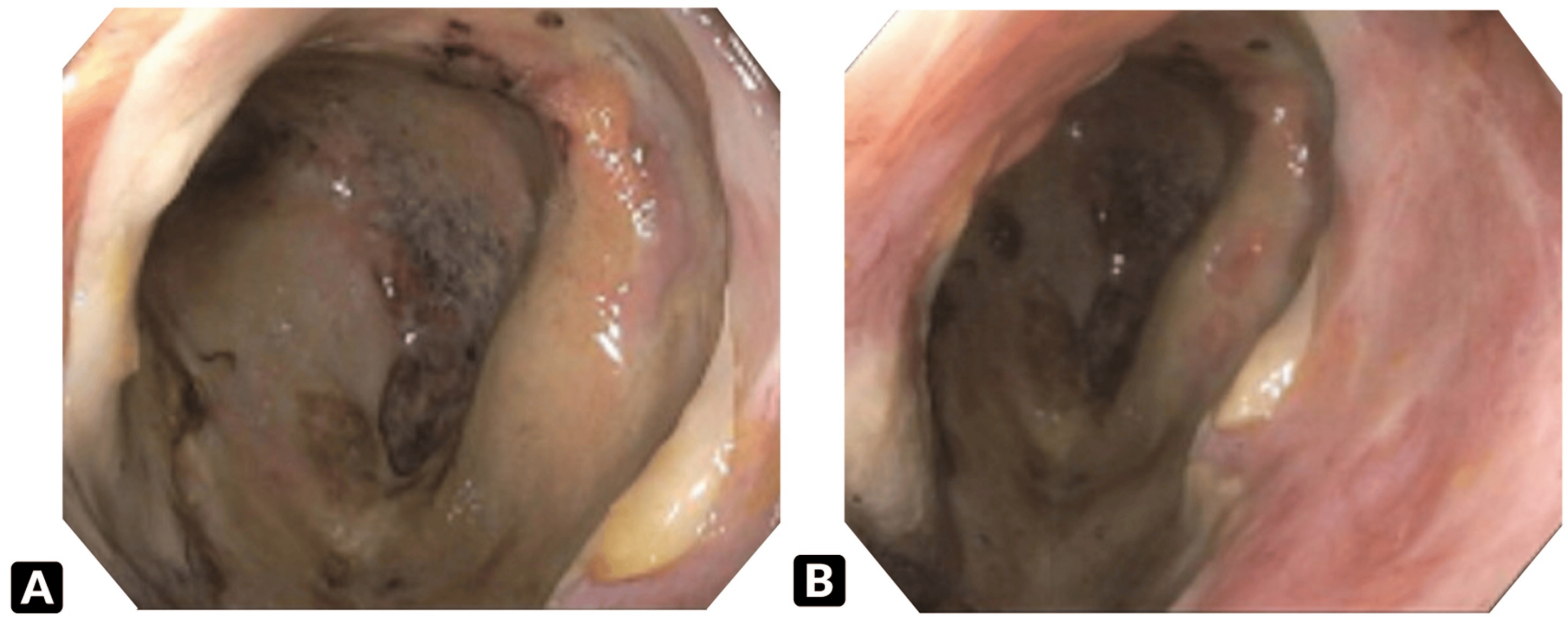
Colonoscopy visualization of the (A) distal transverse colon and (B) descending colon showing severe inflammation and friability due to necroinflammatory exudates.

**Figure 3:**
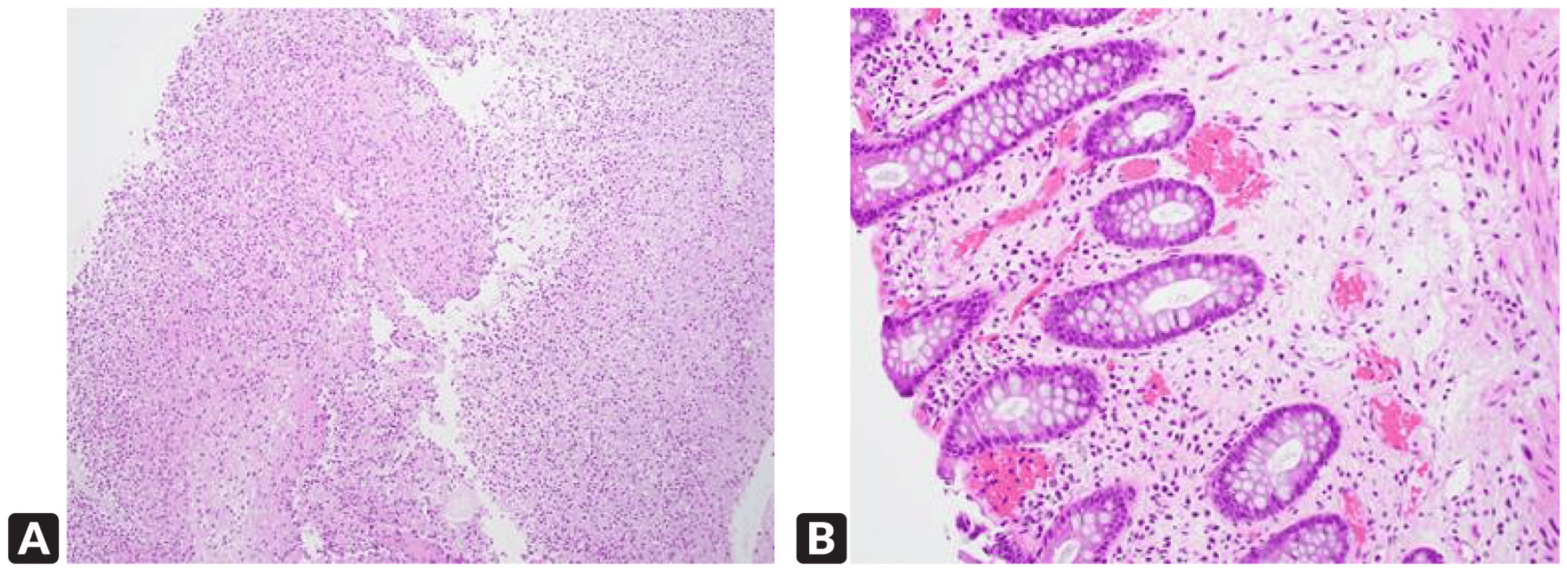
Colonoscopic biopsy specimens. (A) Splenic flexure/transverse colon showing necroinflammatory exudate without viable colonic tissue, consistent with ischemic necrosis. (B) Descending colon showing colonic mucosa with few prominent lymphoid aggregates and mild erosion, without significant inflammation, architectural distortion, dysplasia, or malignancy.

The patient underwent gastroenterological evaluation and an urgent colonoscopy, which revealed severe inflammation with ulceration and friability in the distal transverse colon and splenic flexure. Passage of the scope beyond this region was not possible due to severe narrowing, colonic ulceration, and inflammation (Figure 2). The patient demonstrated only mild inflammation in the sigmoid colon and rectum. Colonic diverticulosis was observed in the descending colon, along with moderate external hemorrhoids. Biopsies from the ulcerated mucosa demonstrated necroinflammatory exudates without viable colonic mucosa (Figure 3).

Approximately 24 hours after a colonoscopy, the patient developed severe abdominal pain accompanied by peritoneal signs. She then underwent an emergency exploratory laparotomy. Intraoperatively, significant adhesions were identified at the splenic flexure, with dense fibrosis involving the ascending colon and appendix. A colonic perforation of the sigmoid colon with significant fecal contamination was also noted. The patient subsequently underwent a subtotal colectomy with ileostomy.

The postoperative course was complicated by a prolonged ileus requiring total parenteral nutrition. Leukocytosis was observed, with a peak white blood cell count of 35,000 cells/µL (reference range, 4.5-11.0x10^3^/µL), which gradually improved while the patient remained on the same empiric IV antibiotic regimen, including meropenem, micafungin, and linezolid.

**Figure 4:**
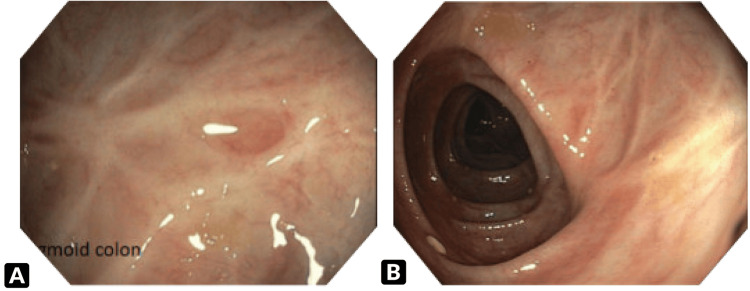
Colonoscopy performed six years prior to the present episode. (A) and (B) show two different areas of the sigmoid colon at 20 and 25 cm with scarred mucosa.

The presentation of the patient was consistent with her prior clinical history. This represents the third hospital admission of the patient for IC. The first episode occurred 14 years earlier, followed by a second one six years later. During the second episode, a CT of her abdomen showed bowel wall thickening and edema with mucosal hyperenhancement of the large intestine extending from the anus to the splenic flexure. A colonoscopy performed five weeks later revealed scarring in the sigmoid colon, presumed to be sequelae of the IC episode (Figure 4).

The patient started sumatriptan therapy for migraine management approximately one year prior to the first hospital admission for IC. She had a known history of chronic migraines treated with oral sumatriptan 100 mg daily and subcutaneous sumatriptan 6 mg as needed, with frequent use of the subcutaneous formulation. Additionally, she had a history of chronic back pain managed with oral diclofenac and tramadol on an as-needed basis.

During the current presentation, her abdominal pain was similar in timing, character, and severity to that experienced during prior episodes of IC.

Over the course of a few weeks, she recovered and was discharged home with instructions regarding ileostomy care. She was advised to permanently discontinue sumatriptan and follow up with her neurologist.

## Discussion

IC classically presents as an acute onset of crampy, left-sided abdominal pain accompanied by an urgent need to defecate [13]. Within 24 hours of abdominal pain, rectal bleeding, manifesting as bright or maroon stools, or bloody diarrhea typically develops [13]. In the present case, the patient presented with acute abdominal pain but had not yet developed overt gastrointestinal bleeding at the time of presentation.

The history and clinical presentation of the patient were important for suspecting IC. Initial laboratory findings were largely unremarkable except for the elevated lactate level. She presented early enough that leukocytosis and metabolic acidosis had not yet developed. Despite this, the patient had severe sepsis, with tachycardia, tachypnea, and hypotension that responded to fluids.

Colonoscopy was performed because it is the gold standard for confirming IC [14]. In this case, the patient developed a colonic perforation 24 hours after colonoscopy. It remains unclear if the perforation was due to the colonoscopy procedure, technical difficulty of the intervention, acute-on-chronic IC, or a combination of these factors. Since colonic perforation is a known risk during colonoscopy in ischemic tissue, precautions are recommended, including limited insufflation and careful inspection [14]. In this case, the scope was not advanced beyond the transverse colon due to severe narrowing. Nonetheless, the patient experienced a colonic perforation requiring subtotal colectomy and ileostomy, followed by recovery over an extended postoperative course.

Drug-induced IC is commonly associated with medications affecting the gastrointestinal and nervous systems [15]. Notable examples include therapies for irritable bowel syndrome, such as alosetron (a serotonin-3 receptor antagonist), tegaserod (a serotonin-4 receptor partial agonist), and eluxadoline (a µ-opioid receptor agonist) [15]. Laxatives are also implicated, as they can induce rapid fluid shifts and colonic hypoperfusion; examples include lubiprostone, lactulose, bisacodyl, and various colonoscopy preparations [15]. Stimulants, including amphetamines and methylphenidate, can compromise colonic blood flow via splanchnic vasoconstriction [16].

Vasoconstrictor medications, including sympathomimetic drugs such as pseudoephedrine and phenylephrine, can induce IC through direct agonistic effects on 𝛼- and 𝛽2-adrenergic receptors [17]. Medications that alter blood pressure and perfusion, including diuretics, beta blockers, and vasodilators such as hydralazine and nitrates, reduce preload and consequently decrease blood supply to the affected organs [17]. Oral contraceptives and estrogen therapy can create a hypercoagulable state implicated in IC [17]. NSAIDs inhibit cyclooxygenase (COX)-2, resulting in an imbalance between vasoconstriction and vasodilation that reduces mucosal blood flow to the colon [18].

Sumatriptan succinate, a serotonin-1 (5-HT1) receptor agonist, has been associated with IC in only several published case reports and small case series [5-12]. The drug binds to intracranial 5-HT receptors, causing vasoconstriction and alleviation of migraine headaches [8]. Among reported cases, some occurred with oral sumatriptan alone, and others involved exposure to both oral and subcutaneous formulations [5-12]. Most patients recovered with supportive care, although outcomes were not uniformly described. The present case was severe, requiring surgical intervention and demonstrating evidence of repeated injury to the colon, as indicated by widespread adhesions observed intraoperatively.

In the present case, sumatriptan was the most likely cause of the IC, as her first hospitalization occurred one year after initiating the medication. However, the patient was also taking 50 mg diclofenac twice daily, another known contributor to IC. It is likely that chronic NSAID use primes the colon, and the addition of sumatriptan further compromises colonic blood flow. This case demonstrates the unfortunate progression to three hospitalizations for IC, ultimately resulting in an ileostomy. During her first two hospitalizations, sumatriptan was not widely reported as a potential cause of IC, underscoring the importance of thorough medication review in patients presenting with IC.

## Conclusions

IC is characterized by reduced blood flow to the colon, which can result from medication use. The 5-HT1 receptor agonist sumatriptan is often prescribed for its vasoconstrictive effects to relieve migraine headaches, but by the same mechanism, it can also compromise colonic perfusion. Therefore, when evaluating patients with acute abdominal pain and a differential diagnosis of IC, thoroughly reviewing their medications is essential. Suspicion should increase when the patient is taking multiple medications associated with IC. Furthermore, patients receiving multiple long-term vasoconstrictive medications should undergo regular outpatient re-evaluation. For example, if a patient complains of recurrent bouts of intermittent abdominal pain and they are on medications at high risk for IC, it is important for the clinician to further delve into the severity, timing relative to medication use, and any associated symptoms like hematochezia or hypotension. Taking a careful and thorough history is a low-cost, high-quality practice that can help prevent potential episodes of IC.
